# A Variation in the Cerebroside Sulfotransferase Gene is Linked to Exercise-Modified Insulin Resistance and to Type 2 Diabetes

**DOI:** 10.1155/2009/429593

**Published:** 2009-07-05

**Authors:** A. Roeske-Nielsen, K. Buschard, J. E. Månson, L. Rastam, U. Lindblad

**Affiliations:** ^1^Bartholin Institute, Rigshospitalet, Ole Maaløvsvej 5, 2200 Copenhagen, Denmark; ^2^Department of Clinical Neuroscience, Sahlgrenska University Hospital, Mölndal, 413 45 Gothenburg, Sweden; ^3^Department of Clinical Sciences, Lund University, 214 01 Malmo, Sweden; ^4^Department of Public Health and Community Medicine/Primary Health Care, The Sahlgrenska Academy, Gothenburg University, 405 30 Gothenburg, Sweden

## Abstract

*Aims*. The glycosphingolipid *β*-galactosylceramide-3-*O*-sulfate (sulfatide) is present in the secretory granules of the insulin producing *β*-cells and may act as a molecular chaperone of insulin. The final step in sulfatide synthesis is performed by cerebroside sulfotransferase (CST) (EC 2.8.2.11). The aim of this study was to investigate whether two single nucleotide polymorphisms (SNP), rs2267161 located in an exon or rs42929 located in an intron, in the gene encoding CST are linked to type 2 diabetes (T2D). *Methods*. As a population survey, 265 male and female patients suffering from T2D and 291 gender matched controls were examined. *Results*. A higher proportion of T2D patients were heterozygous at SNP rs2267161 with both T (methionine) and C (valine) alleles present (49.8% versus 41.3%, *P* = .04). The calculated odd risk for T2D was 1.47 (1.01–2.15, *P* = .047). Among female controls, the homozygous CC individuals displayed lower insulin resistance measured by HOMA-IR (*P* = .05) than the C/T or TT persons; this was particularly prevalent in individuals who exercise (*P* = .03). *Conclusion*. Heterozygosity at SNP rs2267161 in the gene encoding the CST enzyme confers increased risk of T2D. Females with the CC allele showed lower insulin resistance.

## 1. Introduction

Several single nucleotide polymorphisms (SNPs) have been found linked to type 2 diabetes (T2D). These include calpain 10 [1], PPAR*γ* [2], adiponectin [3, 4], and the Kir6.2 subunit of the ATP-sensitive potassium channel [5]. In Icelandic subjects, the transcription factor 7-like 2 (TCF7L2) was found to be strongly associated to T2D [6].

The glycolipid *β*-galactosylceramide-3-*O*-sulfate (sulfatide) modulates cytokine expression [7–9], including TNF-*α* and Interleukin 6 (IL-6) which are known to cause insulin resistance [10]. Sulfatide may also act as a molecular chaperone of insulin, being present in the secretory granules and are secreted together with insulin [11, 12]. Low sulfatide concentration in serum disposes to development of T2D with a relative risk of 2.5, and low serum sulfatide levels are correlated to high insulin resistance [13]. The cerebroside sulfotransferase (CST) (EC 2.8.2.11) catalyzes the final and rate-limiting reaction in sulfatide synthesis: the addition of a sulphate group to galactocylceramide, [14].

The gene encoding CST is located on chromosome 22 (22q12.2). The gene encompasses roughly 22 kb and is comprised of 8 exons, 6 noncoding, and 2 coding exons. The CST cDNA, composed of 1791 bp, encodes a 423-amino acid protein with a predicted type II transmembrane topology and 2 potential N-linked glycosylation sites. At present there are 13 known SNPs in the CST gene but only one is located in a coding exon (exon 7). This variation (rs2267161) causes an amino acid shift at codon 29 alternating between methionine and valine. 

In this study we examined the prevalence of the two SNPs rs2267161 and rs42929 in a group of 265 T2D patients and 291 healthy controls. The individuals were all examined intensively, with several clinical parameters investigated, including BMI, insulin resistance (HOMA-IR), serum sulfatide concentration, and exercise level.

## 2. Methods and Subjects

### 2.1. Subjects

In Skara, Sweden, a large majority of patients with hypertension and/or T2D have attended medical care programs at the local public health center during the last 30 years. During 1992-1993, 400 patients diagnosed with diabetes were given extensively examinations at the health care centre during an extended annual visit, thus participating in the baseline survey of the Skaraborg Hypertension and Diabetes Project, described in detail elsewhere [15]. From the population census register of Skara, 1400 subjects aged ≥40 years were randomly selected, stratified by age and gender to participate in a population survey [16]. From each 10-year category between 40 and 79 years of age, 150 men and women, and in the age group 80 years, 100 men and 100 women were identified. These subjects were invited to the clinic for a clinical examination according to the same protocol as in the 1993-1994 patient survey. 1109 subjects responded and completed the investigation. The Skaraborg Hypertension and Diabetes Project have been approved by the Committee on Research Ethics at the Medical Faculty of Göteborg University, and all participants gave their informed consent. Patients below 75 years of age with T2D were eligible for the present study, and 128 women and 145 men were selected as participants. From the population survey 300 control individuals conforming to the following were selected: <75 years, no evidence of T2D and fasting blood glucose level <5.6 mmol/L, 142 women and 158 men in total. Due to lack of biochemical analysis 4 men (1 patient and 3 controls) were excluded from the study at baseline, and a further 14 were excluded due to failure of genotyping; in total 136 male and 155 female controls partook, along with 139 male and 126 female T2D patients. There was a significant age difference between cases and controls, but within each group the ages of men and women were similar.

### 2.2. Biomedical Data

Body mass index (BMI), blood serum sulfatide concentrations, serum insulin concentration, and fasting blood glucose levels were obtained as described in [13].

### 2.3. Homeostasis Model Assessment Test (HOMA)

Blood samples were drawn in the morning, after 10 hours of overnight fast. Fasting blood glucose was analyzed locally by a modified glucose dehydrogenase method (Hemocue AB, Ängelholm, Sweden). Using the Homeostasis Model Assessment (HOMA) [17, 18] insulin resistance and *β*-cell function were assessed from the fasting glucose and insulin concentrations. Patients with fasting blood glucose levels of less than 3.5 mmol/L and patients in treatment with insulin (65 patients) were excluded from analysis of HOMA values.

### 2.4. Exercise Level

A questionnaire on tobacco use, alcohol consumption, and leisure time physical activity (LTPA) was given to the participants when they came to the study visit. They completed it on site and returned it to the study nurse. LTPA was characterized based on four answer alternatives to the question “How much physical activity do you engage in during your leisure time?” The question referred to the past year and the answer alternatives were as follows. (1) Sedentary leisure time: reading, TV, stamp collecting, or other sedentary activity. (2) Light LTPA: walking, cycling, or other physical activity under at least four hours per week. (3) Moderate LTPA: running, swimming, tennis, aerobic, heavier gardening, or similar physical activity during at least 2 hours a week. (4) Heavy training or competitive sport: heavy training or competitions in running, skiing, swimming, football, and so forth, which is performed regularly and several times a week.

### 2.5. Polymorphism Screening

In total, 265 T2D samples and 291 control samples were tested for the two SNPs (rs2267161 and rs42929). Genotyping was performed using multiplex PCR followed by matrix-assisted laser desorption/ionization time-of-flight mass spectrometry (MALDI-TOF) using SEQUENOM's MassEXTEND assay (Sequenom, San Diego, Calif, USA). Briefly, amplification of genomic DNA, forward and reverse primers specific for the sequence surrounding the two SNPs were constructed, each primer incorporated an hME-10 tag. The hME-10 primers were used in the first multiplex PCR. The amplified, DNA was then treated with shrimp alkaline phosphatase to desphosphorylate any unincorporated dNTPs and subjected to a second multiplex PCR. In the second multiplex PCR, primers specific for the DNA sequences forward or reverse up to the SNPs were used along with terminating dNTPs, specific for the polymorphic nucleotides, creating allele-specific extension products. The molecular weights of the extension products were subsequently analyzed using MALDI-TOF mass spectrometry.

### 2.6. Statistics

Statistical analysis was performed using SPSS 12.0.1 for Windows. Standard methods were used for descriptive statistics. All analyses were adjusted for difference in age and specified also for other covariates. Associations between categorical variables were analyzed by logistic regression and expressed as odds ratios (ORs) with 95% confidence intervals (CIs). Differences in continuous variables were analysed with general linear models (GLMs). All tests were 2-sided, and statistical significance was assumed when *P* < .05.

## 3. Results

Association between two SNPs in the cerebroside sulfotrasferase gene and T2D was examined. Regarding the SNPs rs2267161, the heterozygotic allele C/T was found to be more frequent among the T2D patients than among the controls (49.8% versus 41.3%, *P* = .04) (Table 1). The odd ratio for having T2D increased from 1 (C/C) to 1.59 (confidence interval: 1.07–2.35) with the C/T genotype (*P* = .02), after correcting for age, sex, and physical activity (Table 2).

All participants in the study completed a homeostasis model assessment test (HOMA) for insulin resistance (-IR) and *β*-cell function (-BC). In control females, subjects with the C/C genotype displayed higher insulin sensitivity (*P* = .02) and lower *β*-cell function (*P* = .05) than the C/T or T/T genotypes (Table 3). Further analysis of the lower insulin resistance amongst the female controls revealed that the difference lays entirely amongst women who exercised, (*P* = .03) (Table 3).

Sulfatide levels were significantly lower in the patient group compared to the control group (1.96 versus 2.22), but this difference was not related to any of the genotypes, and no significant difference between the genotypes were observed (see Table 4).

Analysis of rs42929 allele frequency data revealed no significant difference between diabetic patients and controls (Table 5).

## 4. Discussion

Analysis of the allele frequency of the exon located SNPs rs2267161 revealed a higher proportion of T2D patients having the C/T allele than controls. Female controls with the C/C genotype displayed better insulin sensitivity and showed corresponding lower insulin secretion; exercise may be especially beneficial for these individuals as female carriers of the C/C genotype who exercised had lower insulin resistance than carriers of the other genotypes. Interestingly, the C/C allele tended to have a reduced frequency among the female diabetic patients. T2D is correlated to obesity, with women considerably more susceptible to increased risk with increasing BMI [19] and women with T2D experience a 3-fold higher risk of acute myocardial infarct than men with T2D [20]. 

The SNPs rs2267161 causes an amino acid variance between methionine and valine. The predominant genotypes are C/C and C/T coding for valine and valine and methionine. The homozygotic T form (T/T) has the lowest frequency; that is, T is the minor allele at re2267161. Both methionine and valine are overall uncharged and have similar level of hydrophobicity. Valine has a neutral ethyl-side group, whereas methionine has a sulphanyl group. This difference might lead to conformational changes in the CST enzyme depending on which amino acid is used at SNPs rs2267161. The precise functional consequence, at enzyme level, of heterozygoticity at SNPs rs2267161 is not known, but the observed difference between controls and the patient group indicates that increased heterozygoticity confers an increased risk of T2D in the population tested.

Other studies have reported a possible linkage between chromosome 22 and T2D in genomewide linkage analysis using microsatellite markers encompassing the region 22q12.2 where the *CST * gene is located. A UK-based study found a linkage between a loci located at position 22q11 and early onset (<45 years) T2D; the resolution in this study was an average of 9 centimorgans indicating that region 22q12 could be included [21]. A genomic scan of 516 microsatellite markers in 363 Pima Indians revealed a linkage between region 22q12-13 and fasting plasma glucose concentration [22]. In a study including 638 T2D affected African-American sib-pairs, a genomewide multipoint single-locus linkage analysis, the second highest LOD score of 1.33 was seen on chromosome 22 at 32 centimorgans (microsatellite marker D22S685)—region 22q12 [23]. A genomewide linkage search in Canadian Oji-Cree Indians using 190 microsatellite markers (resolution approximately 20 centimorgans), marker D22S683 (encompassing region 22q12) showed significant linkage to T2D [24]. Taken together these reports substantiate our finding that region 22q12 is linked to T2D prevalence.

## 5. Conclusion

Sulfatide affects both the immune system (including low grade inflammation as seen in T2D) and *β*-cell (pato)physiology [25], and the blood concentration of sulfatide is reduced in T2D patients [13]. We here report a connection between a specific genotype and changed insulin resistance in females, linked to exercise level, and a linkage between susceptibility to T2D and a heterozygosity in the coding region of the *CST* gene, the final step in sulfatide synthesis.

## Figures and Tables

**Table 1:** Nucleotide frequencies at SNPs rs2267161. Note difference in frequency of C/T genotype between controls and type 2 diabetic patients. Asterisk indicates *P * < .05.

		rs2267161				
		C/C	C/T	T/T	C	T
Control	Male	0.485	0.382	0.132	0.676	0.324
	Female	0.458	0.439	0.103	0.677	0.323
	Male + Female	0.471	0.412*	0.117	0.677	0.323

DM	Male	0.432	0.482	0.086	0.673	0.327
	Female	0.373	0.516	0.111	0.631	0.369
	Male + Female	0.404	0.498*	0.098	0.653	0.347

**Table 2:** Associations between variations in the CST gene at SNPs rs2267161 and T2D. Associations were estimated using logistic regression and expressed as odds ratios (ORs) with 95% confidence intervals (CIs).

Category (covariates)	Genotype C/C	Genotype C/T			Genotype T/T		
		ORs	CIs	p (C/T versus C/C)	ORs	CIs	p (T/T versus C/C)
Men and women (age)	1	1.47	1.01–2.15	0.047	0.88	0.48–1.62	0.68
Men and women (age, BMI)	1	1.50	1.01–2.24	0.045	0.85	0.45–1.60	0.61
Men and women (age, exercise level)	1	1.59	1.07–2.35	0.02	0.90	0.48–1.69	0.74
Men and women (age, Sulfatid)	1	1.44	0.98–2.12	0.07	0.85	0.46–1.58	0.61
Men and women (age, HOMA-IR)	1	1.32	0.80–2.17	0.28	0.69	0.32–1.49	0.34

**Table 3:** HOMA-IR of controls and type 2 diabetic patients by sex, genotype, and level of exercise. *indicate *P * = .05, ^*#*^indicate *P * = .03.

Gender		Female						Male					
Genotype		C/C			Non-C/C			C/C			Non-C/C		
		Mean	SD	*n*	Mean	SD	*n*	C/C	SD	*n*	Non-C/C	SD	*n*
Controls	All	0.75*	0.20	71	0.97	0.19	84	1.00	0.19	66	1.05	0.25	70
	Exercise	0.52*	0.14	12	1.01	0.29	21	0.87	0.24	21	0.97	0.27	32
	No-exercise	0.80	0.24	59	0.96	0.21	63	1.05	0.21	45	1.11	0.33	38

T2D	All	3.14	0.63	47	3.06	0.63	79	2.49	0.57	60	2.61	0.61	79
	Exercise	2.88	0.68	8	2.21	0.74	4	3.03	0.90	14	2.04	0.63	14
	No-exercise	3.18	0.66	39	3.08	0.65	75	2.37	0.59	46	2.74	0.72	63

**Table 4:** Biomedical data from patients and controls displayed according to genotype at SNPs rs2267161. The serum sulfatide and insulin levels are in log units.

		Genotype C/C				Genotype C/T				Genotype T/T			
		Controls		Type 2		Controls		Type 2		Controls		Type 2	
Male	*n*	66	—	60	—	52	—	67	—	18	—	12	—
		mean	SD	mean	SD	mean	SD	mean	SD	mean	SD	mean	SD
	Age	58.5	10.4	63.9	7.9	57.7	9.6	65.8	6.8	56.2	10.2	66.9	6.1
	BMI	26.3	2.8	28.3	4.1	26.6	3.1	28	4.3	26.1	2.2	28	5.7
	Sulfatid	2.2	0.7	1.9	0.8	2.2	0.8	1.9	0.8	2.0	0.8	2.0	0.8
	Serum insulin	0.7	0.3	0.9	0.4	0.7	0.4	1.0	0.6	0.6	0.4	1.0	0.2
	Fasting blood glucose (mmol/L)	4.6	0.5	8.4	2.3	4.7	0.5	8.4	2.6	4.7	0.4	8.9	2.2

Female	*n*	71	—	47	—	68	—	65	—	16	—	14	—
		mean	SD	mean	SD	mean	SD	mean	SD	mean	SD	mean	SD
	Age	58.9	9.7	65.3	8.1	57.7	10	64.1	8.7	61.1	10.9	68.3	5.4
	BMI	27	5.6	29.2	5.4	26.5	4.5	29.9	4.7	26.3	4.7	31	4.7
	Sulfatid	2.3	0.7	2.2	0.8	2.3	0.7	1.9	0.8	2.1	0.7	2	0.8
	Serum insulin	0.6	0.5	1.0	0.3	0.7	0.3	1.1	0.4	0.8	0.4	0.9	0.3
	Fasting blood glucose (mmol/L)	4.7	0.4	7.9	2.0	4.7	0.5	8.1	2.7	4.8	0.5	9.9	4.0

**Table 5:** The nucleotide frequencies at SNPs rs42929.

		rs42929				
		C/C	C/T	T/T	C	T
Control	Male	0.902	0.090	0.008	0.947	0.053
	Female	0.928	0.072	—	0.964	0.036
	Male + Female	0.916	0.080	0.003	0.956	0.044

DM	Male	0.911	0.089	—	0.956	0.044
	Female	0.894	0.098	0.008	0.943	0.057
	Male + Female	0.903	0.093	0.004	0.949	0.050
